# Association between vaginal microecological alterations and high-risk human papillomavirus infection: a cross-sectional study

**DOI:** 10.3389/fcimb.2025.1618846

**Published:** 2025-07-03

**Authors:** Nanqiu Peng, Jing Xiao, Li He, Li Xie

**Affiliations:** ^1^ College of Medical Technology, Shanghai University of Medicine & Health Sciences, Shanghai, China; ^2^ Department of Research, Zhoupu Hospital Affiliated to Shanghai University of Medicine & Health Sciences, Shanghai, China; ^3^ Department of Microbiological Testing, Xuhui Center for Disease Control and Prevention, Shanghai, China; ^4^ Clinical Laboratory, Wusong Central Hospital, Baoshan District, Shanghai, China; ^5^ Wusong Hospital, Zhongshan Hospital affiliated To Fudan University, Shanghai, China

**Keywords:** no. 101, Tongtai North Road, Baoshan District, Shanghai vaginal microecology, high-risk HPV infection, cervical cancer, correlation study, early Intervention

## Abstract

**Objective:**

This study aimed to investigate the correlation between alterations in the vaginal microecological environment and high-risk human papillomavirus (HR-HPV) infection, thereby providing a theoretical basis for clinical prevention and early intervention.

**Methods:**

A total of 854 patients who visited the gynecology outpatient clinic at Wusong Central Hospital, Baoshan District, Shanghai, between June and December 2023 were included. Vaginal secretions were collected for HPV genotyping and microecological analysis. Patients were categorized into HR-HPV-positive (n=222) and HR-HPV-negative (n=632) groups. The detection rates of various HR-HPV subtypes (HPV 16, 18, 31, 33, 35, 39, 45, 51, 52, 53, 56, 58, 59, 66, 68, 73, 82) were analyzed. Key microecological indicators—including vaginal pH, hydrogen peroxide, leukocyte esterase, sialidase, *Lactobacillus* levels, and vaginal cleanliness—were compared between the two groups, and their predictive value for HR-HPV infection was assessed using ROC curve analysis.

**Results:**

Significant differences were observed between the two groups in terms of vaginal pH, hydrogen peroxide, leukocyte esterase, sialidase activity, *Lactobacillus* abundance, and vaginal cleanliness (all P < 0.05). The ROC curve analysis yielded an AUC of 0.701, indicating moderate predictive value.

**Conclusion:**

Changes in the vaginal microecological environment are significantly associated with HR-HPV infection. Monitoring vaginal microecological indicators may provide adjunctive information for clinical prevention and early intervention, though further prospective studies are needed to establish causality.

## Highlights

- Vaginal microecological imbalance is significantly associated with HR-HPV infection.
- pH > 4.5 and reduced Lactobacillus abundance correlate with higher HR-HPV positivity.
- HR-HPV was most prevalent in women aged 25–40, mainly involving HPV-52 and HPV-58.
- ROC analysis shows vaginal microecological markers moderately predict HR-HPV risk.
- Abnormal vaginal indicators could serve as early warnings for HPV-related cervical lesions.

## Introduction

Cervical cancer is the fourth most common malignancy among women globally, with approximately 600,000 new cases and 340,000 deaths reported in 2020 (Sung et al., 2021). In China, there were about 110,000 new cases and 59,000 deaths in 2021, making it the second leading cause of cancer-related deaths among women (Bruni et al., 2021). Persistent infection with high-risk human papillomavirus (HR-HPV) is the primary etiological factor in the development of cervical cancer (Cho et al., 2024). It is the only cancer with a clearly defined causative agent, with more than 99% of cases linked to persistent HPV infection.

The vaginal microenvironment plays a pivotal role in maintaining cervical health and is closely tied to the host’s immune status and susceptibility to infection. With increasing attention to reproductive health among younger populations, the importance of vaginal microecology has emerged as a key research focus. The vagina hosts a complex microbial ecosystem that exists in dynamic balance with the host and external environment. Disruption of this balance may lead to dysbiosis and a range of gynecological conditions (Lin et al., 2022). Evidence suggests that persistent HR-HPV infection is influenced by alterations in the vaginal microenvironment (Shannon et al., 2017).

This study collected clinical data from gynecological patients at Wusong Central Hospital between June and December 2023. Patients were grouped based on HR-HPV test results. Vaginal cleanliness, fungal presence, pH, hydrogen peroxide, leukocyte esterase, sialidase activity, and *Lactobacillus* levels were assessed and compared. The distribution of HR-HPV subtypes (HPV 16, 18, 31, 33, 35, 39, 45, 51, 52, 53, 56, 58, 59, 66, 68, 73, and 82) was also analyzed. ROC curve analysis was conducted to evaluate the predictive value of vaginal microecological indicators for HR-HPV infection. This study aims to explore the association between vaginal microecology and HR-HPV infection and provide a theoretical foundation for clinical prevention and early intervention.

### Overview of HPV

Human papillomavirus (HPV) is a double-stranded circular DNA virus with over 200 known genotypes, approximately 40 of which are capable of infecting the human genital tract (Muhr and Eklund, 2018). Based on their oncogenic potential, HPV genotypes are classified into low-risk and high-risk types, with HPV-16 and HPV-18 being the most commonly associated with cervical cancer. HPV is primarily transmitted through sexual contact but can also spread via vertical transmission and direct contact. The virus exhibits tissue tropism, specifically targeting epithelial and mucosal tissues, leading to proliferative lesions. Different genotypes vary in their site specificity and pathogenic potential (Varnai et al., 2009).

The E6 and E7 oncogenes of HPV play critical roles in the virus’s carcinogenicity. Their expression is closely associated with the progression of high-grade cervical lesions and can serve as molecular markers in clinical diagnostics (Anastacia and Deanne, 2020). These oncoproteins disrupt cell cycle regulation, induce epithelial cell transformation, and facilitate malignant progression (Yew et al., 2011). Numerous studies have demonstrated a positive correlation between the expression of HPV E6/E7 mRNA and the severity of cervical lesions (Yang et al., 2022). While 50–70% of women may acquire an HPV infection during their lifetime, most low-risk types are cleared by the immune system. In contrast, HR-HPV infections are more persistent and significantly associated with high-grade cervical lesions and cervical cancer (Zhao et al., 2012; Aromseree et al., 2014).

Currently, two primary strategies are employed to control HPV infection: early detection and treatment, and preventive vaccination. The bivalent (Cervarix), quadrivalent (Gardasil), and nonavalent (Gardasil 9) vaccines are prophylactic and effectively prevent infection by HR-HPV types, particularly HPV-16 and HPV-18. Compared with the bivalent and quadrivalent vaccines, the nonavalent vaccine covers a broader range of high-risk genotypes—including HPV 31, 33, 45, 52, and 58—preventing nearly 90% of HPV-related cervical cancers and genital warts (Boda et al., 2018).

China has made significant strides in promoting HPV vaccination to reduce HPV infection and cervical cancer incidence. However, disparities in healthcare access, especially in underdeveloped regions, continue to pose challenges. For individuals already infected with HR-HPV, prophylactic vaccines offer limited benefit; thus, timely clinical intervention remains essential for disease prevention.

HR-HPV persistence is influenced by a combination of host and environmental factors (Shannon et al., 2017). Recent studies have highlighted the pivotal role of the vaginal microenvironment in modulating the risk and progression of cervical cancer (Wei et al., 2022).

### Vaginal microenvironment

The vaginal microenvironment comprises the local immune system, anatomical structures, endogenous microbial flora, and endocrine regulation (Yew et al., 2011). Under normal physiological conditions, these elements maintain a dynamic but stable balance. However, this balance is highly susceptible to external and internal influences, including immunosuppression, aging, sexual activity, pregnancy, and childbirth.

Dysbiosis of the vaginal microbiota is associated with increased incidence of vaginitis and cervical disorders. Fortunately, such imbalances are potentially reversible with early detection, improved immunity, and appropriate treatment. If unaddressed, they may evolve into various vaginal pathologies, such as bacterial vaginosis (BV) (Sekaran et al., 2023).

BV has been strongly associated with HR-HPV infection (Lu et al., 2015). It is characterized by an overgrowth of anaerobic bacteria such as *Gardnerella vaginalis*, and a concomitant decline in protective *Lactobacillus* species. *Lactobacillus* is the predominant commensal in the healthy vaginal flora. It helps maintain an acidic vaginal pH, inhibits pathogenic colonization, and supports mucosal immune function. A decrease in *Lactobacillus* levels disrupts this acidic milieu, elevates vaginal pH, and fosters microbial imbalance—ultimately increasing the risk of BV.

Research indicates that microecological disturbances are closely linked to the severity of cervical intraepithelial neoplasia. Parameters such as hydrogen peroxide concentration, vaginal cleanliness, and pH are considered reliable indicators for predicting cervical lesion severity (Li et al., 2020). A growing body of evidence supports a significant association between persistent HR-HPV infection and vaginal microecological imbalance (Trama et al., 2022).

This study aims to systematically explore the relationship between changes in vaginal microecology and HR-HPV infection. By elucidating this correlation, we seek to provide objective evidence to inform early clinical intervention and preventive strategies.

## Materials and methods

### Study population

#### Participants

**Figure 1:**
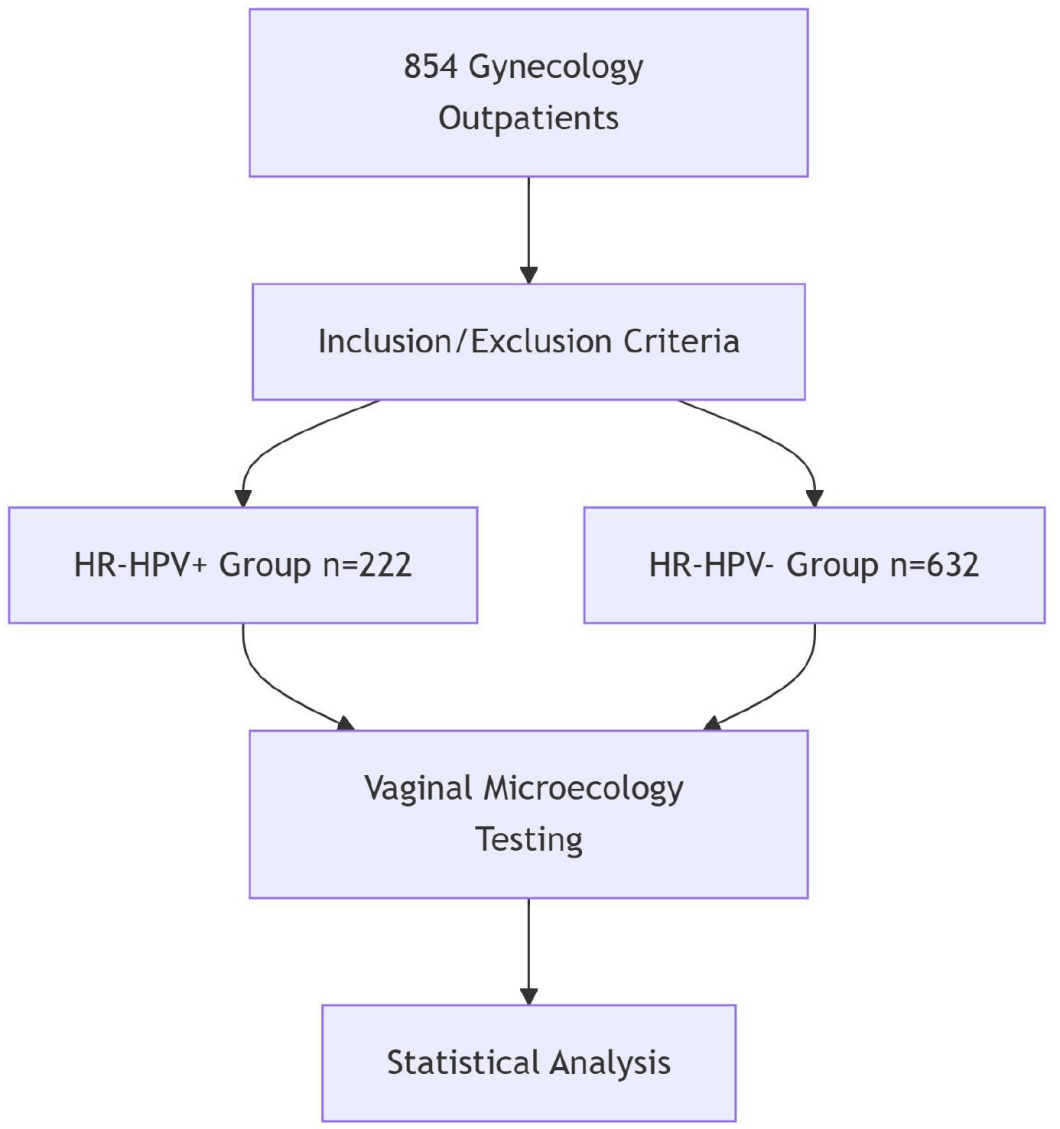
Study flowchart.

This study enrolled 854 patients who visited the gynecology outpatient clinic at Wusong Central Hospital in Shanghai’s Baoshan District between June and December 2023(**Figure 1**).

##### Inclusion criteria

Participants met the following conditions:

1. History of sexual activity;
2. No sexual activity within 7 days prior to sampling;
3. No use of medications affecting vaginal secretions within the preceding 24 hours;
4. No vaginal douching, bathing, or topical medications within 24 hours before testing;
5. No history of radiotherapy or chemotherapy;
6. No hepatic or renal dysfunction;
7. No cardiovascular or hematologic diseases.

##### Exclusion criteria

Patients were excluded if they met any of the following:

1. Pregnancy or lactation;
2. History of malignancies;
3. History of HIV infection;
4. Presence of autoimmune disorders;
5. Comorbid diabetes, hypertension, or cardiac disease;
6. Uterine endometrial or pelvic malignancies;
7. Use of exogenous hormones or history of hormone abuse.

### Experimental procedures

#### Sample collection

All specimens were collected by experienced gynecologists during the non-menstrual period. Sterile, dry equipment free of lubricants or chemicals was used. After inserting a speculum to expose the vaginal canal, a sterile cotton swab was inserted into the posterior fornix and gently rotated for 10–20 seconds to collect cervical and vaginal secretions. The swab, now visibly moistened, was placed into a sterile tube containing transport medium. Samples were stored at –70°C and brought to room temperature for one hour before testing.

#### Vaginal microecological assessment

Samples were analyzed using a dry chemistry reagent kit for bacterial vaginosis and an automated vaginal secretion analyzer. The following indicators were assessed:

1. Vaginal Cleanliness: Evaluated based on the presence of bacilli, cocci, leukocytes, and epithelial cells. Grades I and II were considered normal; grades III and IV were deemed abnormal.

2. pH Value: A normal vaginal pH ranges from 3.8 to 4.5. A pH above 4.5 was considered indicative of dysbiosis.

3. Microbial Functional Markers:

- Hydrogen Peroxide (H_2_O_2_): Negative results suggest adequate Lactobacillus presence; positive results indicate dysbiosis.
- Leukocyte Esterase: Positivity suggests vaginal inflammation.
- Sialidase: Positivity indicates the potential presence of bacterial vaginosis.

4. Lactobacillus Quantification: Assessed microscopically under oil immersion. Abundance was graded:

- 1+ (fewer than 5 per field),
- 2+ (5–20),
- 3+ (20–150),
- 4+ (more than 150).

Grades 3+ to 4+ were considered normal; 1+ to 2+ were considered abnormal.

### HPV genotyping

1. Nucleic Acid Extraction: DNA was extracted using a magnetic bead-based kit (Jiangsu Shuoshi Biotech Co., Ltd.) and the SSNP-9600A automatic nucleic acid extraction instrument. Each sample (200 μL) was loaded into the extraction plate and processed according to standard procedures.

2. Genotyping Procedure: HPV subtyping was performed using a fluorescent quantitative PCR system (SLAN-96P, Shanghai Hongshi) and a commercial HPV DNA genotyping kit targeting 17 high-risk subtypes (HPV 16, 18, 31, 33, 35, 39, 45, 51, 52, 53, 56, 58, 59, 66, 68, 73, 82). Probes specific to each subtype were labeled with fluorescent dyes (FAM, HEX, ROX). Detection was based on amplification of the L1 gene region. Fluorescence signal interpretation was automated, with results reviewed manually to ensure accuracy.

3. Diagnostic Criteria:

- Absence of all HR-HPV subtypes = HR-HPV negative;
- Detection of one subtype = single infection;
- Detection of ≥2 subtypes = multiple infection.

### Statistical analysis

All data were analyzed using SPSS 24.0. Categorical variables were presented as percentages and compared using the chi-square test. Continuous variables were expressed as mean ± standard deviation ( $\bar{x}$ ± s) and compared using the t-test. A P-value < 0.05 was considered statistically significant. Multivariate logistic regression analysis was used to evaluate the association between vaginal microecological factors and HR-HPV infection. Receiver operating characteristic (ROC) curves were plotted, and the area under the curve (AUC) was calculated to assess the predictive value of each indicator.Variables with P < 0.1 in univariate analysis were included in the multivariate model. Univariate and multivariate logistic regression results are presented in **Tables 1**, **2**.

**Table 1:** Univariate logistic regression analysis.

Indicator	OR (95% CI)	P-value
pH >4.5	2.12 (1.04-4.32)	0.039
H_2_O_2_ Positive	2.23 (1.31-3.79)	0.003
Leukocyte esterase Positive	4.26 (2.85-6.37)	<0.001
Sialidase Positive	4.69 (1.51-14.52)	0.007
Lactobacillus 1+~2+	1.49 (1.10-2.01)	0.010
Cleanliness III°-IV°	2.34 (1.69-3.24)	<0.001

**Table 2:** Multivariate logistic regression analysis.

Indicator	aOR (95% CI)	P-value
pH >4.5	1.82 (0.87-3.81)	0.112
H_2_O_2_ Positive	1.78 (1.02-3.12)	0.042
Leukocyte esterase Positive	3.76 (2.46-5.74)	<0.001
Sialidase Positive	3.92 (1.22-12.57)	0.022
Lactobacillus 1+~2+	1.32 (0.95-1.84)	0.098
Cleanliness III°-IV°	1.97 (1.38-2.82)	<0.001

## Results

### Baseline characteristics

A total of 854 patients aged 17 to 85 years were included in the study. Among them, 222 were HR-HPV positive, with an age range of 18 to 78 years and a mean age of 40.56 ± 14.11 years. The HR-HPV negative group consisted of 632 patients, aged 17 to 85 years, with a mean age of 38.77 ± 11.33 years. There was no statistically significant difference in age between the two groups (P > 0.05).

### Distribution of HR-HPV infections

Among the 854 participants, 222 were positive for HR-HPV, yielding an overall infection rate of 26.00%. A total of 17 HR-HPV genotypes were identified. Single-type infections were found in 172 cases (77.48%), with the most frequently detected genotypes being HPV-52 (21.51%), HPV-58 (12.21%), and HPV-39 (11.63%). Multiple-type infections were observed in 50 cases (22.52%) (**Table 3**).

**Table 3:** Distribution of HR HPV genotypes.

HPV genotype	Cases (n)	Detection Rate (%)
Single-type infections		
16	10	5.81
18	8	4.65
31	4	2.33
33	5	2.91
35	4	2.33
39	20	11.63
45	2	1.16
51	9	5.23
52	37	21.51
53	8	4.65
56	11	6.40
58	21	12.21
59	16	9.30
66	7	4.07
68	8	4.65
73	1	0.58
82	1	0.58
Total	172	77.48
Multiple-type infections		
HPV genotype	Cases (n)	Detection Rate (%)
Involving HPV-16	21	42.00
Not involving HPV-16	29	58.00
Total	50	22.52

Multiple infection refers to detection of two or more HR-HPV genotypes.

### Age-specific distribution of HR-HPV infections

Participants were stratified into four age groups to assess age-related patterns of HR-HPV infection. The highest infection rate (48.65%) was observed in the 25–40-year age group, followed by the 41–55-year group (22.07%) (**Table 4**).

**Table 4:** Analysis of HR HPV infection in different age groups.

Age Group	n	HRHPV negative	HRHPV positive				Infection Rate (%)
			16	18	Other types	Multiple Infection	
<25	36	11	1	1	20	3	11.26
25-40	510	402	7	6	78	17	48.65
41-55	217	168	1	0	32	16	22.07
>55	91	51	2	1	23	14	18.02
Total	854	632	11	8	153	50	

### Vaginal microecological indicators and HR-HPV infection

Following HPV genotyping, all samples underwent vaginal microecological evaluation. Results demonstrated significantly higher rates of abnormal indicators in the HR-HPV-positive group compared to the HR-HPV-negative group, including elevated vaginal pH, hydrogen peroxide positivity, leukocyte esterase, sialidase, lower *Lactobacillus* abundance, and poorer vaginal cleanliness (P < 0.05) (**Tables 1**, **2**, **5**).

**Table 5:** Comparison of vaginal microecological indicators between groups.

Indicator	HR HPV Negative (n = 632)	HR HPV Positive (n=222)	χ²	P-value
PH ≤ 4.5	54 (8.5%)	9 (4.1%)	4.848	<0.05
PH>4.5	578 (91.5%)	213 (95.9%)		
H_2_O_2_ Negative	100 (15.8%)	17 (7.7%)	9.264	<0.05
H_2_O_2_ Positive	532 (84.2%)	205 (92.3%)		
Esterase of white blood cells was negative	577 (91.3%)	158 (71.2%)	55.491	<0.05
Esterase of white blood cells is positive	55 (8.7%)	64 (28.8%)		
Sialidase negative	627 (99.2%)	214 (96.4%)	8.669	<0.05
Sialidase positive	5 (0.8%)	8 (3.6%)		
Lactobacillus 1+~2+	263 (41.6%)	114 (51.4%)	6.318	<0.05
Lactobacillus 3+~4+	369 (58.4%)	108 (48.6%)		
Cleanliness I°-II°	307 (48.6%)	64 (28.8%)	26.074	<0.05
Cleanliness III°-IV°	325 (51.4%)	158 (71.2%)		

### Predictive value of vaginal microecological indicators for HR-HPV

Multivariate logistic regression was applied to assess the predictive power of vaginal microecological indicators for HR-HPV infection. An ROC curve was generated based on the model’s predicted probabilities.

**Figure 2:**
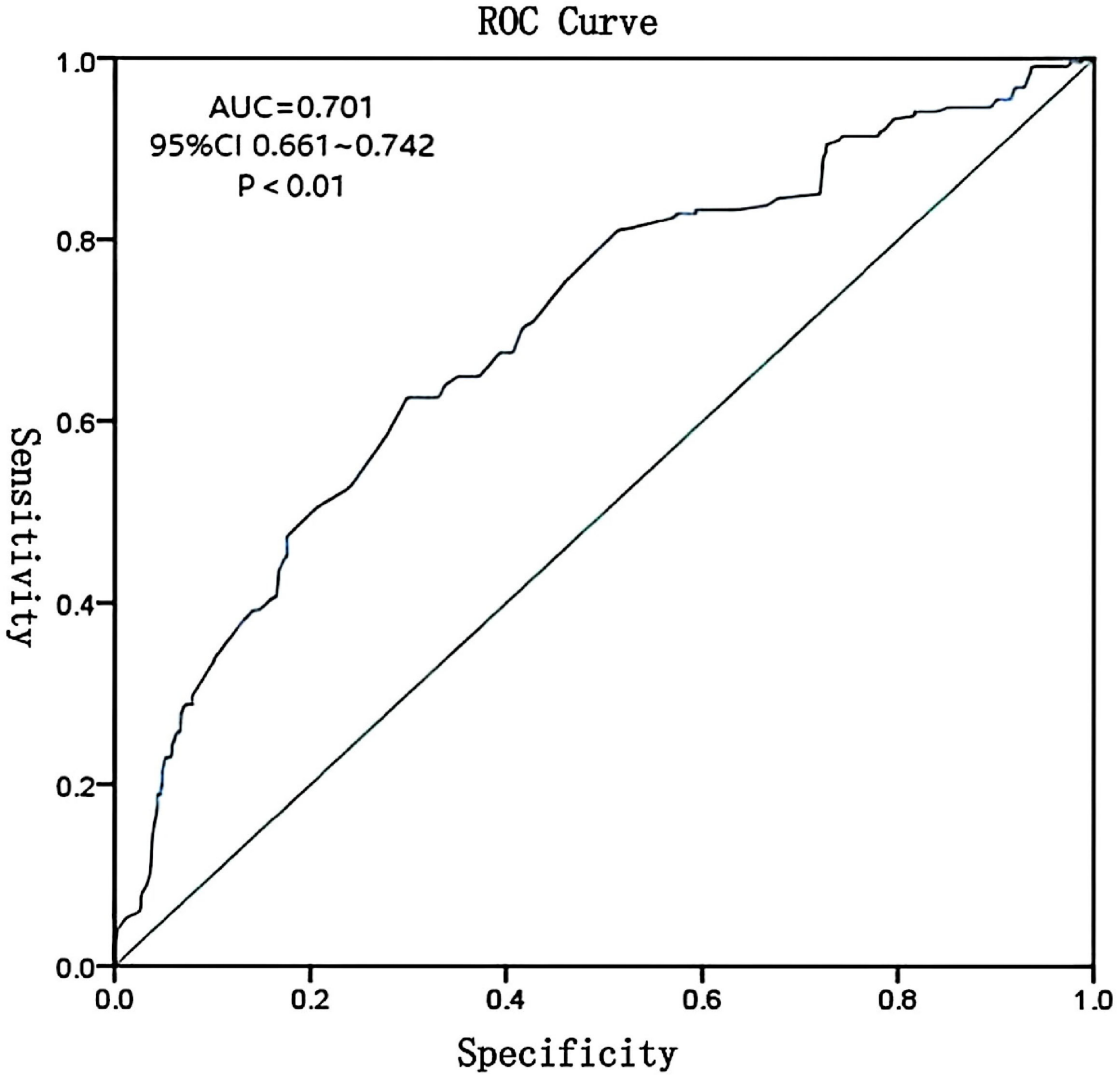
ROC curve for composite model of vaginal microecological indicators predicting HR-HPV infection. AUC = 0.701, 95% CI: 0.661–0.742.

The ROC curve was constructed using predicted probabilities from the multivariate logistic regression model (combined indicators). The area under the curve (AUC) was 0.701 (95% CI: 0.661-0.742), with a sensitivity of 62.6% and specificity of 70.1% at the optimal cutoff value of 0.32, indicating moderate predictive accuracy (**Figure 2**).

## Discussion

Cervical cancer remains one of the most prevalent malignancies among women globally, ranking second in incidence among female cancers. According to the U.S. Centers for Disease Control and Prevention (CDC), at least 50% of sexually active individuals will acquire an HPV infection at some point in their lives (Trama et al., 2022). As the only cancer with a well-established viral etiology, cervical cancer is highly preventable through vaccination and early screening. Consequently, strategies for effective prevention and intervention have become a critical focus in gynecological research.

Growing evidence suggests a strong association between vaginal microecological disturbances and the acquisition and persistence of HPV infection (Yuejuan et al., 2019). Once vaginal microecological homeostasis is disrupted, the resulting dysbiosis increases susceptibility to various infections, including HR-HPV, thereby influencing the progression of cervical disease.

The anatomical proximity of the vagina to the anus and urethra, coupled with hormonal fluctuations during menstruation and pregnancy, renders the vaginal environment particularly vulnerable to microbial imbalance. During these periods, reduced immune function can increase infection risk. Vaginal discharge analysis—a routine clinical tool—enables detection of both morphological and functional changes, providing valuable information for the diagnosis of reproductive tract infections and cervical lesions.

With advances in laboratory diagnostics, many hospitals now utilize reagent-based assays for comprehensive assessment of vaginal secretions. Measurements such as pH, hydrogen peroxide concentration, leukocyte esterase, and other biochemical indicators improve the accuracy of diagnoses related to vaginitis and HPV infection (Dun et al., 2023).

### HR-HPV infection status

HPV infection rates vary widely by geographic region and population demographics. Factors such as economic development, lifestyle, and awareness levels affect regional genotype distribution. Data from mainland China show HR-HPV prevalence ranging from 17.1% to 22.1%, with East China reporting an average rate of 19.3%. The most common HR-HPV types are HPV 52, 16, 53, 58, and 51; less frequently detected types include HPV 35, 45, 73, and 82 (Zeng et al., 2022). In Shanghai, the prevalence is approximately 18.81%, with HPV 52, 16, 58, 53, and 39 being the most common genotypes (Li et al., 2022).

In this study, the HR-HPV prevalence was 26.00%, slightly higher than the regional average. The dominant genotypes were HPV 52, 58, 39, 59, and 56, while HPV 73, 82, 45, 35, and 31 were least frequently detected. This elevated prevalence may be attributed to the study’s limited sample size and population mobility. These findings are generally consistent with data from Changning District (21.59%) (Hou et al., 2022), but higher than those reported in Zhongshan (12.04%) (Wang and Zeng, 2023) and lower than in Jiangsu Province (30.68%) (Fei et al., 2015), highlighting regional variability in HR-HPV distribution.

### Age-specific HR-HPV infection patterns

Studies have shown that factors such as early onset of sexual activity, high coital frequency, and multiple abortions increase the risk of persistent HR-HPV infection (Zhao et al., 2012). Infection peaks typically occur in two age groups: 17–25 years and 40–45 years. In this study, the highest infection rate was observed in women aged 25–40 years (48.65%), likely due to higher sexual activity and increased likelihood of pregnancy or abortion during this life stage—both of which can disrupt the vaginal microenvironment (Xiong et al., 2018). However, younger women often possess stronger immune responses, increasing the probability of spontaneous HPV clearance.

The second highest infection rate (22.07%) occurred among women aged 41–55 years. This may be due to reduced immune competence, lower education levels, poor personal hygiene practices, and limited awareness of HPV prevention (Westrich et al., 2017). As estrogen levels decline with age, *Lactobacillus* abundance decreases, further impairing vaginal microbial balance (Yoshikata et al., 2022). Since menopause typically occurs around age 50, this age group may experience estrogen deficiency-related dysbiosis, increasing their susceptibility to HR-HPV infection.

### Impact of vaginal microecology on HR-HPV infection

Vaginal microecological disruption plays a crucial role in HR-HPV persistence. Comparative analysis between HR-HPV-positive and -negative groups revealed that pH > 4.5, hydrogen peroxide, leukocyte esterase, and sialidase positivity, as well as reduced *Lactobacillus* levels and poor cleanliness grades (III°–IV°), were significantly more prevalent among infected individuals.

Under normal conditions, the vaginal pH is maintained at 3.8–4.5, primarily by *Lactobacillus* species, which synthesize hydrogen peroxide to inhibit pathogen proliferation and support immune function. When *Lactobacillus* levels decline, hydrogen peroxide production decreases, disrupting this protective mechanism. Consequently, pathogenic organisms may proliferate, mucosal immunity is compromised, and the risk of persistent HR-HPV infection increases—eventually contributing to cervical neoplasia (Shannon et al., 2017). These findings affirm the association between vaginal dysbiosis and increased HPV susceptibility.

Vaginal cleanliness grading reflects microbial composition and inflammation. Grades III°–IV° indicate diminished *Lactobacillus* and overgrowth of opportunistic pathogens, a state conducive to persistent HPV infection and cervical disease progression (Liu et al., 2024). The high prevalence of these grades in HR-HPV-positive individuals further supports this link.

Leukocyte esterase, a neutrophil-derived enzyme, indicates inflammation but is not pathogen-specific. Elevated sialidase levels often signal bacterial vaginosis. In this study, both markers were significantly elevated in the HR-HPV-positive group, consistent with prior research. These findings suggest that coexisting vaginal infections—potentially due to poor hygiene or immune suppression—may facilitate HR-HPV persistence (Chao et al., 2021).

### Predictive value of vaginal microecological indicators

Multivariate logistic regression analysis demonstrated that a combination of vaginal pH, hydrogen peroxide, leukocyte esterase, sialidase, *Lactobacillus* levels, and cleanliness grades could predict HR-HPV infection with moderate accuracy. The ROC curve yielded an AUC of 0.701, with 62.6% sensitivity and 70.1% specificity, indicating that these indicators have meaningful clinical value in early risk assessment. However, the moderate AUC suggests limited predictive power when used in isolation. Integration with additional clinical markers may enhance predictive accuracy.

### Study limitations

This study has several limitations. First, the relatively short sampling period and limited sample size (854 cases) may reduce generalizability. Second, vaginal microecology is influenced by numerous factors including genetics, lifestyle, and hormonal fluctuations, which were not fully controlled. Additionally, all samples were collected from outpatient settings, where undiagnosed reproductive tract infections or long-term medication use may have confounded results. Future studies should incorporate larger, more diverse cohorts and stricter inclusion criteria to validate and expand on these findings.Fifth, as a cross-sectional study, our design cannot establish a causal relationship between vaginal microecological alterations and HR-HPV infection. Longitudinal studies are needed to track the temporal sequence of events and determine whether dysbiosis precedes HR-HPV infection or vice versa.

## Conclusion

This study analyzed 854 female patients and found that HR-HPV infection predominantly involved single-type infections, with HPV-52 being the most commonly detected genotype. Comparative analysis revealed that HR-HPV-positive individuals exhibited significantly higher rates of abnormal vaginal microecological indicators—including elevated pH, increased hydrogen peroxide, leukocyte esterase, and sialidase positivity, as well as decreased *Lactobacillus* abundance and poorer vaginal cleanliness—compared to HR-HPV-negative individuals.

These findings demonstrate a significant association between vaginal microecological imbalances and HR-HPV infection. Monitoring vaginal microecological parameters may provide adjunctive information for risk assessment. However, given the cross-sectional design, further prospective studies are necessary to confirm clinical utility for early intervention strategies.

## Data Availability

The original contributions presented in the study are included in the article/supplementary material. Further inquiries can be directed to the corresponding author.
